# Lung toxicity of lomustine in the treatment of progressive gliomas

**DOI:** 10.1093/noajnl/vdac068

**Published:** 2022-05-10

**Authors:** Corinna Seliger, Christina Nürnberg, Wolfgang Wick, Antje Wick

**Affiliations:** Department of Neurology and National Center for Tumor Diseases, University Hospital Heidelberg, Heidelberg, Germany; Department of Neurology and National Center for Tumor Diseases, University Hospital Heidelberg, Heidelberg, Germany; Clinical Cooperation Unit Neurooncology, German Cancer Consortium (DKTK), German Cancer Research Center (DKFZ), Heidelberg, Germany; Department of Neurology and National Center for Tumor Diseases, University Hospital Heidelberg, Heidelberg, Germany; Clinical Cooperation Unit Neurooncology, German Cancer Consortium (DKTK), German Cancer Research Center (DKFZ), Heidelberg, Germany; Department of Neurology and National Center for Tumor Diseases, University Hospital Heidelberg, Heidelberg, Germany

**Keywords:** CCNU, glioma, lung toxicity, recurrent glioblastoma

## Abstract

**Background:**

Pulmonary fibrosis is a rare, but dangerous side effect of CCNU (lomustine). CCNU is a frequently used chemotherapeutic agent in the setting of recurrent or progressive glioblastoma. At present, CCNU is also administered in patients with newly diagnosed gliomas in combination with temozolomide. There is only little evidence if, and how, lung function should be monitored on treatment with CCNU.

**Methods:**

We retrospectively collected data on patient characteristics, lung function analyses, and relevant toxicities among 166 brain tumor patients treated with CCNU at a German University Hospital and National Cancer Center.

**Results:**

The patient collective mainly included patients with recurrent glioblastoma who received a mean number of 2.64 ± 1.57 cycles. There was overall no statistically significant change in parameters of pulmonary restriction among patients treated with CCNU. On an individual patient basis, a >10% decrease in the absolute vital capacity was primarily seen in patients with prior lung diseases and smokers. Other severe toxicities mainly included thrombocytopenia, leukopenia, nausea, and vomiting.

**Conclusions:**

Our findings support to limit lung function analyses on CCNU to patients with gliomas and pulmonary risk factors. However, all patients should be closely followed for clinical symptoms of pulmonary restriction.

**Key Points**

- We analyzed serial lung function analyses in glioma patients treated with CCNU.
- The risk of pulmonary function decrease is generally dispensable in these patients.
- Regular lung function analysis should be restricted to pulmonary at-risk patients.

**Importance of the Study**

CCNU is one of the standard therapies available for patients with recurrent glioblastoma and used with increasing frequency also for patients with newly diagnosed glioblastoma and hypermethylated MGMT promoter. While CCNU is widely used, there is uncertainty amongst therapists about the meaningfulness of repetitive lung function analyses for early detection of lung fibrosis—a rare but serious side effect of CCNU. As the first to analyze data on sequential lung function analyses in this patient collective, we aim to provide a guide in the pulmonary surveillance of patients treated with CCNU. Ultimately, we present evidence that there is a rationale for repeated pulmonary function testing only for patients at risk with prior lung diseases or other concurrent diseases. We do not see the necessity for repetitive pulmonary testing in patients that do not fulfill these criteria.

Diffuse gliomas are the most common primary brain tumors in adults.^1^ They can be subdivided into different CNS (Central Nervous System) WHO (World Health Organization) grades and according to the presence versus absence of an IDH (isocitrate dehydrogenase) mutation.^2^ IDH-mutated oligodendrogliomas (CNS WHO grades 2 and 3) and astrocytomas (CNS WHO grades 2–4) share a better prognosis as compared to IDH-wildtype gliomas including glioblastoma (CNS WHO grade 4), H3.3 G34-mutant diffuse hemispheric gliomas (CNS WHO grade 4), and H3 K27M-altered diffuse midline gliomas (CNS WHO grade 4).^2^

The standard of care for gliomas includes maximal safe surgical resection or biopsy followed by radio- and/or chemotherapy.^3,4^ There are only few chemotherapeutic agents in the treatment of gliomas, as many substances are not sufficiently able to cross the blood–brain barrier. Whereas temozolomide is mostly used in the primary setting of glioblastoma,^5^ CCNU (chloroethyl-cyclohexyl-nitrosourea or equally called lomustine) is frequently used in the primary therapy of lower grade oligodendrogliomas and astrocytomas,^6–8^ and in recurrent glioblastomas.^9^ Furthermore, the addition of CCNU to radiochemotherapy with temozolomide may prolong survival of patients with newly diagnosed methyl-guanine-methyl-transferase (MGMT) promoter methylated glioblastomas.^10^ CCNU may also be combined with procarbazine and vincristine in the so-called “PCV” regimen,^6,7,11^ used as monotherapy or in combination with other agents such as etoposide or teniposide.^9,12^ CCNU is a member of the group of nitrosoureas, which mainly act by alkylation of DNA and RNA, crosslinking of DNA and carbamoylation of amino acids.^13^ MGMT may revert CCNU-induced lesions such as the building of *O*^6^-chloroethylguanine.^9^ Common toxicities of CCNU include nausea and vomiting, myelosuppression with especially thrombocytopenia and leukopenia, increased liver enzymes or rarely secondary tumors.^13^ Pulmonary fibrosis is a rare, but dangerous side effect of nitrosoureas.^13^ The absence of relevant rates of lung fibrosis in clinical trials led to the assumption that lung function does not have to be routinely monitored in asymptomatic patients receiving CCNU.^9^ However, in clinical routine, there is an uncertainty if lung function should be regularly monitored or not with different local standards.

We therefore performed a cohort study based on data from a large German Brain Tumor Center to explore how lung function is affected by CCNU in the routine treatment of brain tumor patients.

## Materials and Methods

### Patient Cohort

The patient cohort consisted of brain tumor patients treated with CCNU at the University Hospital and National Cancer Center Heidelberg, Germany. Patients were included if they were treated with CCNU in the time between 01.03.2014 and 01.08.2020 and if they had at least 1 lung function analysis. Data on sex, age, tumor histology, IDH-mutational status, MGMT promoter methylation status, number of recurrences, CCNU regimen, dexamethasone treatment, Karnofsky Performance Status (KPS), concurrent diseases, and smoking status were retrieved from database review. The appropriate institutional review board approved the project. Informed consent was not necessary as all data were anonymized and retrospectively collected from routine patient data.

### Lung Function Analyses

Lung function was measured by spirometry at the Department of ENT (Ear-Nose-Throat) at the University Hospital Heidelberg. Vital capacity (VC), forced vital capacity (FVC), and forced expiratory volume (FEV1) was measured in liters and additionally given in %-of normal before the start of CCNU treatment and before cycles 3 and 5, where available.

### Assessment of Toxicities

Data on severe toxicities (grade 4) according to the Common Terminology Criteria for Adverse Events (CTCAE)^14^ were derived from database review.

### Statistical Analyses

GraphPad Prism version 9.3.1 was used for all statistical analyses. Since the same cohort of patients were tested repetitively, we assumed dependent data and consequently applied paired calculations. Here, all the values that were only tested at 1 time point, but not at the other, are eliminated, that is, the sample size is reduced accordingly. Statistical significance was assessed by the 2-sided Student’s *t*-test for normally distributed data. Otherwise a Wilcoxon matched-pairs signed rank test was used for nonnormal distributions which was the case for the majority of data. All available data were depicted as column bar graphs with mean + SD. The level of significance was set at *P* < .05.

## Results

Our cohort consisted of 166 patients treated with CCNU at the University Hospital Heidelberg. Most patients in our cohort were treated for glioblastoma (70.48%) and received CCNU in combination with etoposide (96.34%). Mean age of patients at the time of their lung function analysis was 54.56 ± 12.90 years. A KPS above 70% was present in 69.28% of patients and 64.46% of patients were men. Most patients (90.96%) were nonsmoker and 63.86% of patients took steroids. The number of recurrences was as follows: 36.75% (first recurrence), 27.10% (second recurrence), 22.29% (third recurrence), 9.64% (fourth recurrence), and 4.22% (fifth recurrence). The MGMT promoter was hypermethylated in 43.37% of patients, not hypermethylated in 31.33% and unknown in 25.30%. IDH showed a wildtype status in 54.22% of patients, 33.73% of patients were IDH-mutant and 12.05% had an unknown IDH-mutational status (Table 1). The mean number of CCNU cycles was 2.64 ± 1.57.

**Table 1.** Patient characteristics

Variable	Characteristic	Frequency (*N* = 166)	Percentage
Sex	Male	107	64.46
	Female	59	35.54
Age group	≤55	88	53.01
	>55	78	46.99
Histology	Glioblastoma	117	70.48
	Oligodendroglioma, IDH-mutant and 1p19q codeleted, CNS WHO grade 2	5	3.01
	Oligodendroglioma, IDH-mutant and 1p19q codeleted, CNS WHO grade 3	2	1.20
	Astrocytoma, IDH-mutant, CNS WHO grade 2	6	3.61
	Astrocytoma, IDH-mutant, CNS WHO grade 3	15	9.04
	Others	21	12.65
IDH mutation	IDH-mutant	56	33.73
	IDH-wildtype	90	54.22
	Not determinable	20	12.05
MGMT promoter methylation	Methylated	72	43.37
	Nonmethylated	52	31.33
	Unknown	42	25.30
Number of recurrences	1	61	36.75
	2	45	27.10
	3	37	22.29
	4	16	9.64
	5	7	4.22
CCNU regimen	CCNU monotherapy	1	0.60
	CCNU + etoposide	160	96.34
	CCNU + vincristine	1	0.60
	Unknown	4	2.41
Dexamethasone treatment	Yes	106	63.86
	No	59	35.54
	N/A	1	0.60
KPS	≤70	52	31.33
	>70	112	69.28
	Unknown	2	1.20
Concurrent disease	None	49	29.52
	Epilepsy	72	43.37
	Atrial fibrillation	6	3.61
	Arterial hyertension	39	23.49
	DVT/pulmonary embolism^a^	8	4.82
	Asthma	7	4.22
	Others^b^	27	16.27
Smoking status	Smoker	15	9.04
	Nonsmoker	151	90.96

KPS, Karnofsky Performance Status.

^a^One patient suffered both from DVT (deep vein thrombosis) and pulmonary embolism.

^b^Including 1 patient with pulmonary fibrosis.

**Figure 1.**
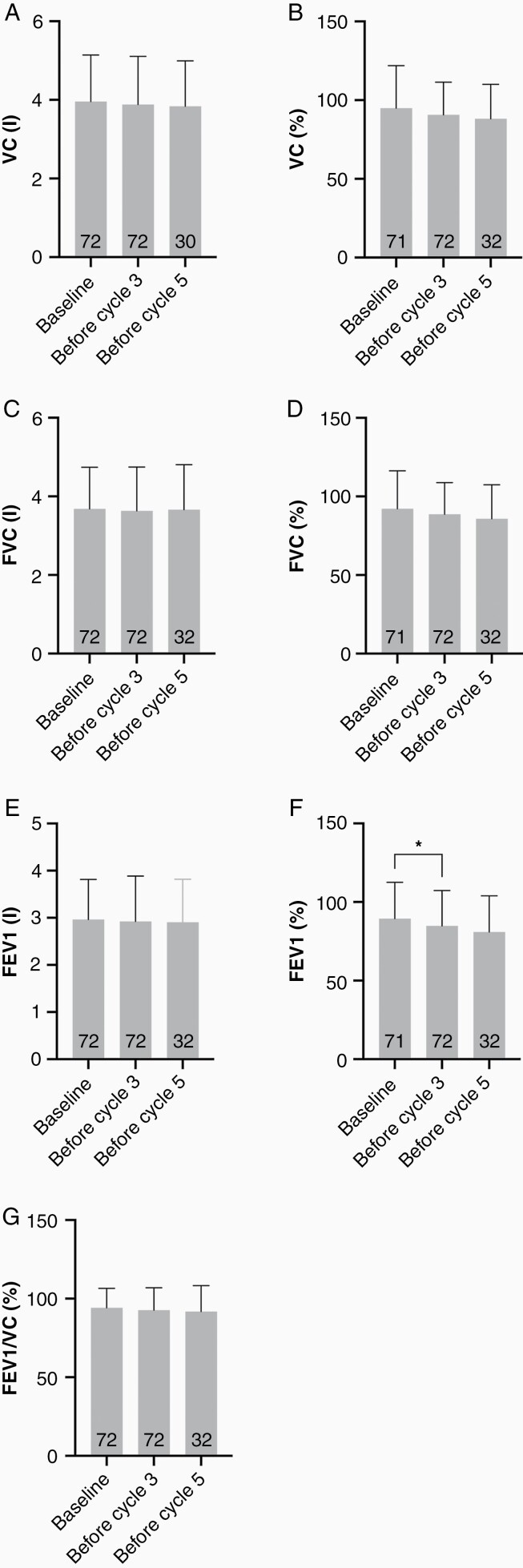
Lung function analysis. Vital capacity (VC) was assessed in liters (a) and %-of normal (b) at baseline, before cycle 3, and before cycle 5. Forced vital capacity (FVC) in liters (c) and %-of normal (d), forced expiratory volume (FEV1) in liters (e) and %-of normal (f), and forced expiratory volume/vital capacity in %-of normal (g) were also analyzed at baseline, before cycle 3, and before cycle 5.

Seventy-two patients received more than 1 pulmonary function test. There was no significant change of the mean VC in liters or %-of normal at baseline, after 2 cycles and after 4 cycles of CCNU. Correspondingly, the mean FVC in liters or %-of normal remained stable at the 3 respective time points. Whereas mean FEV1 in liters was not materially changed from baseline after 2 cycles and after 4 cycles of CCNU, mean FEV1 in %-of normal decreased slightly with 89.44 ± 23.08 at baseline, 84.85 ± 22.50 after 2 cycles and 81.00 ± 22.88 after 4 cycles. Mean FEV1/VC (%) was comparable at the 3 time points. The only analysis that reached statistical significance was the comparison between FEV1% at baseline and after 2 cycles of CCNU (Figure 1 and Table 2).

**Table 2.** Mean results of lung function analyses

Parameter	Mean value ± SD at baseline	Mean value ± SD before cycle 3	Mean value ± SD before cycle 5
VC (l)	3.96 ± 1.19	3.88 ± 1.22	3.84 ± 1.15
VC (%)	94.98 ± 27	90.68 ± 20.75	88.18 ± 21.85
FVC (l)	3.68 ± 1.06	3.63 ± 1.11	3.66 ± 1.14
FVC (%)	92.15 ± 24.19	88.64 ± 20.2	85.82 ± 21.66
FEV1 (l)	2.96 ± 0.85	2.92 ± 0.96	2.90 ± 0.96
FEV1 (%)	89.44 ± 23.08	84.85 ± 22.5	81.00 ± 22.88
FEV1/VC (%)	94.20 ± 12.38	92.64 ± 14.26	91.82 ± 16.54

FEV1, forced expiratory volume; FVC, forced vital capacity; VC, vital capacity.

We further looked at lung function analysis of glioma patients on an individual basis and studied 9 patients with a >10% decrease between the baseline and sequential absolute VC. About one-third (33.33%) of those patients were smokers and 55.55% of those patients had prior or concurrent lung diseases (pulmonary embolism, lung fibrosis, pneumonia). About one-third of patients (33.33%) with a >10% decrease in lung function had no lung diseases, but other concurrent diseases including arterial hypertension, breast cancer, and seizures. There was 1 patient (11.11%) with a significant decrease in lung function, but without other concurrent diseases, except for nicotine abuse.

Among the full cohort of 166 patients, 86.75% of patients had no toxicity greater than CTCAE grade 3. Grade 4 thrombocytopenia was present in 0.6% of patients, 2.41% of patients had grade 4 leukopenia, 0.6% had severe nausea and vomiting, or other severe toxicities, respectively (Table 3).

**Table 3.** Toxicities other than lung toxicity >CTCAE grade 3

Toxicity >CTCAE grade 3	Frequency (*N* = 166)	Percentage
Anemia	0	0.00
Leukopenia	4	2.41
Thrombopenia	1	0.60
Pancytopenia	0	0.00
Nausea/vomiting	1	0.60
Other toxicity >CTCAE grade 3	1	0.60
No toxicity >CTCAE grade 3	144	86.75
Unknown	15	9.04

CTCAE, Common Terminology Criteria for Adverse Events.

## Discussion

In this cohort of 166 patients with recurrent and progressive glioma receiving CCNU, there was overall no increase in parameters of pulmonary restriction. Patients with >10% decrease in baseline VC had either concurrent lung diseases including nicotine abuse or other concurrent diseases, which may reduce the general physical fitness and resilience against toxic side effects rather than exerting direct effects on lung function. Other severe toxicities mainly included thrombocytopenia, leucopenia, and nausea and vomiting.

The first reports on severe pulmonary toxicity in patients treated with nitrosoureas date back to the 1970s.^15–18^ Most of those studies reported on interstitial pulmonary fibrosis in patients with repeated administrations of carmustine (BCNU = 1,3-bis-(2chloroethy1)-1-nitrosourea) at high-cumulative doses (1.200–1.500 mg/m² body surface), but there were also reports on patients that developed irreversible lung fibrosis after a single administration of BCNU.^19^ BCNU treatment led to diffuse alveolar damage and organizing interstitial pneumonia and fibrosis as seen for other agents like bleomycin.^16,20^ Lung toxicity (pulmonary infiltrates and/or fibrosis) has been reported to occur in up to 30% of patients in the official drug information of BCNU.^21^ Despite those observations, BCNU has been used in the routine treatment of glioma and pulmonary fibrosis was seen as rarely as in 0.6% of chemotherapy-naive patients in a retrospective cohort study.^22^ In studies based on patients with extracranial tumors, use of nitrosoureas significantly increased the risk of pulmonary fibrosis and risk estimates were especially high, when patients also received chest irradiation.^23,24^ If affected, lung fibrosis is a life-threatening condition^22,25^ and may occur up to 17 years after BCNU administration.^26^

Whereas the majority of studies on lung toxicity are based on BCNU, several studies also reported on lung fibrosis after administration of nimustine (ACNU),^25^ fotemustin,^27^ CCNU,^28^ and novel nitrosoureas such as SarCNU.^29^

The frequency of lung toxicity after use of CCNU ranges between 1/1.000 and 1/10.000 patients.^30^ In clinical trial populations using CCNU as standard of care in recurrent glioblastoma, lung fibrosis has not been a toxicity of concern.^31–33^ Grade 3 and 4 respiratory toxicities according to the CTCAE^14^ in patients treated with CCNU were not reported in the BELOB trial.^32^ In the REGAL trial^31^ and INTELLANCE-2 trial,^33^ grade 3 and 4 lung toxicity in patients taking CCNU were limited to pulmonary embolism. In the primary therapy of glioblastoma, 3% of patients treated with the combination of CCNU and temozolomide developed grade 3 and 4 lung infections in the CeTeG trial, whereas 2% of patients in the temozolomide arm developed grade 3 and 4 lung infections. There were no reports on pulmonary fibrosis in the CCNU and temozolomide arm.^10^ Several studies explored PCV in the primary therapy of lower grade glioma.^6,7,11^ In the study conducted by Cairncross et al., PCV therapy led to grade 3 and 4 lung toxicity in 4% of patients during chemotherapy and 1% of patients after chemotherapy as compared to 0% of patients treated with radiotherapy only.^6,34^ Lung toxicity was not specified for lung fibrosis. In the study led by Van Den Bent et al.,^35^ and Buckner et al.,^8^ no grade 3 and 4 lung toxicity was reported.

Reports on toxicity of PCV or CCNU in the routine clinical setting are sparse,^36,37^ but important, as patients treated within clinical trials may be fitter and therefore may experience less side effects. Interestingly, although some groups evaluated PCV toxicity in routine clinical settings, lung toxicity was not even reported.^36,37^ Our data showed that there was overall no decrease in parameters of pulmonary restriction such as the absolute or relative VC or FVC. Decreases in the VC were seen in patients with prior lung diseases, nicotine abuse and other concurrent diseases. The small, but statistically significant decrease in FEV1 is a marker of pulmonary obstruction, but not restriction and therefore not associated with pulmonary fibrosis. Regular assessment of lung function by spirometry increases the duration and expenses of practice visits, and alternatives to CCNU in the recurrent setting of glioblastoma are rare. Therefore, regular monitoring of lung function by spirometry may not be justified in patients with recurrent glioblastoma. However, clinicians should be aware of patients at increased risk of pulmonary restriction and routinely ask and clinically examine for pulmonary symptoms in patients treated with CCNU.

Other severe toxicities (CTCAE grade 4) reported in our cohort are in line with published studies mainly including thrombocytopenia, leucopenia, and emesis.^9^

Our study has several limitations. Data were collected retrospectively and numbers of patients with several lung function analyses were limited. Most patients in the analysis were diagnosed with recurrent glioblastoma with limited patient prognosis, therefore long-term pulmonary toxicities may have been lost and results cannot be translated to younger people with lower grade tumors. Most patients were treated with CCNU and etoposide and results may not be translated to patients treated with PCV. The combination of nitrosoureas and etoposide/teniposide may be used in the treatment of recurrent glioblastomas (adapted from the German Neuro-Oncology Working Group 01 trial^12^), but standard of care at tumor recurrence is poorly defined^9^ and different CCNU regimens are used according to local treatment standards. The mean number of administered cycles of CCNU in the present data set was low when compared to other studies.^8^ Therefore, the cumulative dose of CCNU may not have been high enough to induce lung toxicity. We did not perform sequential chest X-ray examinations or blood gas analyses, which are part of the CTCAE criteria for grade 1–3 lung fibrosis.

In conclusion, our findings support to limit lung function analyses under CCNU to patients at risk with either prior lung diseases or other concurrent diseases including nicotine abuse or symptoms of worsening lung function.
